# Letter to the Editor concerning ‘Personal versus therapist perioperative music intervention: a randomized controlled trial’

**DOI:** 10.1097/JS9.0000000000001614

**Published:** 2024-05-09

**Authors:** Jingzhi Wang, Yulu Rao, Yuanmin Zhang

**Affiliations:** a School of Clinical Medicine, Jining Medical University; b Department of Joint Surgery and Sports Medicine, Affiliated Hospital of Jining Medical University, Jining Medical University, Jining, Shandong, People’s Republic of China

*Dear Editor,*

We read and discussed with great interest a paper by Jiang *et al*.^1^ entitled ‘Personal versus therapist perioperative music intervention: a randomized controlled trial.’ This study included 229 patients scheduled for elective surgery. They were randomly divided into doctoral-designed treatment (TT), personal music intervention (PI) and control patients with standard PACU care without music (Control) three groups, and the study found that compared with the TT group, the PI group was able to improve systolic blood pressure, anxiety, nausea, emesis, and overall satisfaction, but not sleep quality. However, music intervention was able to significantly prevent anxiety and emesis and improve overall satisfaction compared with the control group. We are very interested in the study of music therapy for perioperative patients, so we have some questions to ask the authors.

Firstly, the study of Bradt *et al*.^2^ found that the frequency of music therapy will affect the treatment of patients, and different intervention times can produce different treatment effects. However, the frequency of perioperative music intervention was not unified in this study, which may affect the accuracy of the study results. Secondly, polysomnography (PSG) is the gold standard for recording and monitoring sleep quality^3^. In this study, the Pittsburgh sleep quality index (PSQI) and QON five-grade scoring were used to evaluate the sleep quality of patients, and no music intervention was found to improve sleep quality. As an objective sleep quality evaluation tool, PSG can be used to evaluate the sleep quality of patients, which can improve the reliability and rigor of the research results. In addition, Palmer *et al*.^4^ found that music intervention had a good sedative effect on elderly patients undergoing transurethral resection of the prostate (TURP) under spinal anesthesia. However, our study included only patients 18–60 years of age who were scheduled for elective surgery, so the findings are not generalizable to patients 80–90 years of age.

Finally, we thank the authors once again for their efforts in this study and look forward to their guidance on our questions.

## Ethical approval

None.

## Consent

None.

## Sources of funding

This article was approved by NSFC cultivation project of Jining Medical University (No.JYP2019KJ33).

## Author contribution

J.W. and Y.R.: wrote the manuscript and Y.Z.: revised it.

## Conflicts of interest disclosure

There are no conflicts of interest.

## Research registration unique identifying number (UIN)

None.

## Guarantor

Yuanmin Zhang.

## Data availability statement

None.

## Provenance and peer review

None.
